# AIM2 as a putative target in acute kidney graft rejection

**DOI:** 10.3389/fimmu.2022.839359

**Published:** 2022-09-30

**Authors:** Nathália Franchon Marques Tejada, João Vitor Ziroldo Lopes, Luis Eduardo Duarte Gonçalves, Izabela Mamede Costa Andrade da Conceição, Glória Regina Franco, Bruno Ghirotto, Niels Olsen Saraiva Câmara

**Affiliations:** ^1^ Laboratory of Transplantation Immunobiology, Institute of Biomedical Sciences, Department of Immunology, University of São Paulo, São Paulo, Brazil; ^2^ Laboratory of Biochemical Genetics, Department of Biochemistry and Immunology, Institute of Biomedical Sciences, Federal University of Minas Gerais, Belo Horizonte, Brazil; ^3^ Laboratory of Clinical and Experimental Immunology, Department of Nephrology, Escola Paulista de Medicina, Federal University of São Paulo, São Paulo, Brazil

**Keywords:** kidney transplant, acute rejection (AR), AIM2 inflammasome, biomarker, bioinformatics, inflammasome

## Abstract

Acute rejection (AR) is a process triggered *via* the recognition of grafted organ-derived antigens by the immune system, which could present as a life-threatening condition. In the context of a kidney transplant, despite improvement with immunosuppressive therapies, AR maintains a significant incidence of 10%, and currently available drugs generally act in similar and canonical pathways of lymphocyte activation. This prompted the research for different approaches to identify potential novel targets that could improve therapeutic interventions. Here, we conducted a transcriptome analysis comparing groups of acute rejection (including T cell-mediated rejection and antibody-mediated rejection) to stable grafts that included differentially expressed genes, transcription factor and kinase enrichment, and Gene Set Enrichment Analysis. These analyses revealed inflammasome enhancement in rejected grafts and AIM2 as a potential component linked to acute rejection, presenting a positive correlation to T-cell activation and a negative correlation to oxidative phosphorylation metabolism. Also, the AIM2 expression showed a global accuracy in discerning acute rejection grafts (area under the curve (AUC) = 0.755 and 0.894, p < 0.0001), and meta-analysis comprising different studies indicated a considerable enhancement of AIM2 in rejection (standardized mean difference (SMD) = 1.45, [CI 95%, 1.18 to 1.71]), especially for T cell-mediated rejection (TCMR) (SMD = 2.01, [CI 95%, 1.58 to 2.45]). These findings could guide future studies of AIM2 as either an adjuvant target for immunosuppression or a potential biomarker for acute rejection and graft survival.

## 1 Introduction

Kidney transplant has become the key therapeutic strategy for organ failure, especially in the context of chronic diseases such as diabetes, lupus, polycystic kidney disease, and hypertension (1–3). Since its beginning in the 20th century, advances in surgical and conservation techniques allowed an increase in its numbers worldwide: from 2000 to 2020, the total sum of kidney transplants has drastically increased (from 23,084 to above 100,000), as well as its rate per population (from 9.8 to 14.01 per million) (4). These values, however, are below the general demand (5, 6), which prompted a continuous search for strategies that allowed a greater acquisition and offer of organs—such as the use of expanded criteria donor and a central distribution strategy—as well as interventions that reduce organ rejection and dysfunction (7–9).

During transplantation, the kidney is subjected to ischemia (10). This phenomenon leads to graft inflammation and necrosis, resulting in the release of damage-associated molecular patterns (DAMPs) (11). After graft reperfusion, the presence of DAMPs triggers an immune response from the host, leading to immune cell migration and adaptive response, which could contribute to acute rejection (AR) (12, 13).

AR can manifest as an early (<12 months) or late (>12 months) complication of kidney transplant (14). It can be divided according to Banff criteria histopathological findings in antibody-mediated rejection (ABMR), T cell-mediated rejection (TCMR), and borderline for TCMR. TCMR is initiated *via* the presentation of donor alloantigens to recipient T lymphocytes, leading to T-cell (CD4+ and CD8+) and natural killer recruitment to the allograft, accumulating in kidney tubules, the interstitium, and vascular areas. This process leads to histologic features that allow its classification *via* Banff criteria in interstitial inflammation, tubulitis, or intimal arteritis (15). The activation of T helper cells leads to the release of soluble factors that support the activation of B cells that could trigger ABMR. ABMR is a severe form of acute rejection and can occur with or without TCMR. After recognition and presentation of recipient HLA and DAMPs, T helper cells interact with B cells, leading to antibody affinity maturation and plasma cell activation. In the graft, antibodies formed to activate the complement pathway and trigger neutrophil recruitment, leading to the endothelial lesion and cell death (16, 17). In the Banff criteria, ABMR is characterized for vascular inflammation—utilized as evidence of acute tissue injury—current antibody and endothelial interaction and serologic markers of donor-specific antibodies (18).

A common mechanism that connects both TCMR and ABMR is the innate inflammatory response triggered upon recognition of DAMPs and mismatched HLA. In this sense, one potential component of innate immunity that has been recently put in perspective in the context of transplant rejection is the inflammasomes (19). Inflammasomes are multimeric protein complexes that are activated *via* PRR located in the cytosol, recruiting and activating caspase precursors, leading to IL-1β and IL-18 production and consequent induction of proinflammatory immune responses (20).

Another current therapy target is immunomodulation *via* metabolism reprogramming. Studies regarding T-cell metabolism showed that glycolysis is linked to an increase in inflammation, with Th1 and Th17 mostly relying on glycolytic activity and the mammalian target of rapamycin complex I (MTORC1) pathway to shape an effector response (21, 22). However, OXPHOS and lipid metabolism are linked to immunosuppressive response, with the products of these metabolic pathways being used to produce immunosuppressive cytokines as well as the persistence of memory T cells and generation of T regulatory cells (Tregs) (21–24). For graft rejection, animal studies have shown that the inhibition of glycolysis could prevent or delay rejection by blocking T-cell polarization and increasing relative Treg frequency (25).

Here we performed a transcriptomics analysis, where we aimed to evaluate non-canonical genes and pathways linked to AR in the context of human kidney transplant in comparison to a non-rejecting group. Our analysis indicated possible associations related to the regulation of the inflammatory response and metabolism that could drive graft rejection, which might pave the way for new therapeutic approaches to the patients.

## 2 Materials and methods

### 2.1 Microarray data curation

A query was made in the National Center for Biotechnology Information (NCBI) open-source Gene Expression Omnibus (GEO2R) platform (https://www.ncbi.nlm.nih.gov/geo/) for the term “kidney transplant” filtering study type to “expression profile by array” and limiting results to “*Homo sapiens*” organism. Inclusion criteria comprised kidney tissue samples of acute rejection datasets that presented stable grafts as control. Exclusion criteria included co-transplanted receptors, concomitant chronic rejection, previous meta-analysis, and studies evaluating treatment interventions.

### 2.2 Differentially expressed gene filtering

Three selected datasets (GSE25902, GSE36059, and GSE129166), all from Affymetrix Human Genome U133 2.0 Array platform, were employed for initial screening. Acute rejection samples were compared to stable grafts by applying the GEO2R online tool (26), which enables to identify differences in gene expression using GEOquery and limma (Linear Models for Microarray Analysis) R packages (27). The threshold for differentially expressed genes (DEGs) was set for a false discovery rate (FDR) <0.05, considering upregulated the genes with logFC > 1 and downregulated the ones presenting logFC < −1. Subsequently, to visualize DEG similarity between datasets, the UpsetR graph and Venn diagram were performed using *UpsetR v.1.4.0* (28) and *VennDiagram v.1.6.20* (29) packages for R.

### 2.3 Transcription factor and kinase enrichment analyses

The X2K Appyter (Expression2Kinases) tool was used to perform transcription factor and kinase enrichment analyses based on the DEG list obtained. This tool predicts upstream regulatory networks associated with inputted sets of genes. Discrete query gene sets were compared first to ChEA3 libraries of transcription factor target gene sets assembled from orthogonal “omics” datasets. Afterward, ChEA3 results were put through a protein–protein interaction database to determine the transcription factor intermediate protein interactors. Finally, protein interactors were compared to the KEA3 background database—which contains measured and predicted kinase–substrate interactions, kinase–protein interactions, and interactions supported by co-expression and co-occurrence data—to determine which kinases may be most closely associated with the transcription factor intermediate protein interactors. The top 10 transcription factors and kinases were selected from the enrichment analysis and displayed in the bar charts (**Supplementary Figure 2**).

### 2.4 Gene enrichment and functional analyses

#### 2.4.1 Common differentially expressed gene enrichment analysis

To characterize commonly upregulated genes in all three datasets (GSE25902, GSE36059, and GSE129166), we used Venn diagram intersections obtained at jvenn: an interactive Venn diagram viewer (inra.fr) (30). We performed ClusterProfiler analysis in R software version 4.0.2. Out of 52 commonly upregulated genes, we used “*ClusterProfiler*” to generate enrichment plots of gene ontology analysis through “*DOSE*”, “*enrichplot*”, and “*reactomePA*” packages (31). We generated color-themed dot plots of enriched terms and used them to illustrate statistical biological processes, cellular components, molecular function, and overall representation. We used FDR < 0.05 as a criterion to select enriched terms.

#### 2.4.2 Gene set enrichment analysis

To perform this analysis, we used the computational program Gene Set Enrichment Analysis (GSEA) (gsea-msigdb.org) (32) to upload expression values obtained from the raw data of each dataset. We phenotyped samples as “Acute Rejection” and “Rejection Free” for comparisons and gene set as the permutation type. We selected enriched pathways based on the following criteria: nominal p-value <0.05 and FDR < 0.25. To perform correlation analysis, we set *AIM2* gene as the phenotype label to compare with the whole dataset. We also altered the metric for ranking genes for Pearson’s correlation to provide statistical analysis. We selected the top 10 negatively correlated genes present in the oxidative phosphorylation term to be plotted. We generated heatmaps using the Morpheus software (https://software.broadinstitute.org/morpheus) (33).

### 2.5 Clinical outcome measurements

To assess *AIM2* accuracy in identifying acute kidney rejection, the receiver operating characteristic (ROC) curve with multivariate logistic regression was performed on four chosen datasets (GSE25902, GSE36059, GSE129166, and GSE21374). With the use of a cutoff value comprising 72.55% sensitivity and 54.11% specificity for failure risk, samples of GSE21374 were classified into low or high *AIM2* expression, aiming to further compare graft survival by the Kaplan–Meier method and log-rank test. Graphs were performed in GraphPad Prism 8.0.1 (GraphPad Software, San Diego, CA, USA), and differences were considered statistically significant for p-value <0.05.

For external validation, a meta-analysis was designed to include 16 of the 18 filtered studies, excluding two of *Zhejiang university human 449 oligonucleotide array* platforms that did not identify *AIM2* gene. Groups had standardized mean difference (SMD) comparison evaluated with random-effects models using the *meta v.4.19-1* R package, as previously described for microarray samples (34, 35). Heterogeneity evaluation was performed with *InfluenceAnalysis* of *dmetar v.0.0.9000* package (36).

### 2.6 Statistical analysis

Statistical analysis was performed to *AIM2* level expression and correlations in GraphPad Prism 8.0.1 (GraphPad Software, San Diego, CA, USA). For expression comparison, unpaired t-test and one-way ANOVA followed by the Bonferroni test were used, respectively, for two and three or more group analyses. The plots represented mean ± standard deviation (SD). For correlation measurement, data were initially sorted according to normality distribution using the Kolmogorov–Smirnov test. In normalized samples, correlations were accessed using Pearson’s parametric test, while non-normalized groups were analyzed with non-parametric Spearman’s rank correlation. Correlations’ results were represented by dot plot using the “*ggplot2*” package (37). Results were considered statistically significant when the p-value <0.05.

## 3 Results

### 3.1 Study curation and common differentially expressed gene identification

A systematic search was performed on the Gene Expression Omnibus platform identifying 18 datasets of acute kidney rejection (**Supplementary Figure 1**). Of them, three did not differentiate acute rejection subtypes, three presented exclusively TCMR grafts, one presented just ABMR rejection, one presented only borderline change, and 10 embraced distinct proportions of TCMR, ABMR, borderline, mixed, or non-specific rejections (**Table 1**). To avoid distinct readouts according to platforms and to embrace common processes of different rejections types, three studies (GSE25902, GSE36059, and GSE129166) were selected for initially DEG analysis, including, respectively, the datasets with a higher sample number of TCMR, ABMR, or borderline rejection.

**Table 1:** Studies’ demography.

	GSE1563	GSE9489	GSE9493	GSE14328	GSE21374	GSE25902	GSE36059	GSE48581	GSE34437	GSE52694	GSE72925	GSE76882	GSE106675	GSE114712	GSE129166	GSE147089	GSE174020
Year	2004	2009	2009	2010	2010	2011	2013	2013	2014	2015	2016	2016	2018	2018	2019	2020	2021
**Stable graft**	*n = 10*	*n = 8*	*n = 21*	*n = 18*	*n = 207*	*n = 24*	*n = 281*	*n = 222*	*n = 16*	*n = 14*	*n = 45*	*n = 99*	*n = 10*	*n = 8*	*n = 60*	*n = 168*	*n = 12*
*Donor type*																	
LD	5 (50%)	N/A	5 (23.8%)	N/A	N/A	N/A	N/A	N/A	N/A	N/A	N/A	N/A	N/A	0 (0%)	N/A	33 (19.6%)	N/A
DD	5 (50%)	N/A	2 (0.95%)	N/A	18 (8.7%)	N/A	N/A	N/A	N/A	N/A	N/A	N/A	N/A	8 (100%)	N/A	135 (80.3%)	N/A
*Gender: male/female*																	
Donor	N/A	N/A	N/A	41%/59%	30%/70%	N/A	N/A	N/A	50%/50%	N/A	N/A	N/A	N/A	86%/14%	N/A	48.7%/51.3%	N/A
Recipient	70%/30%	86%/14%	67%/33%	56%/44%	60%/40%	N/A	N/A	N/A	75%/25%	N/A	N/A	N/A	N/A	75%/25%	N/A	67.1%/42.9%	N/A
*Age (years)*																	
Donor	N/A	45.2 ( ± 15.4)	N/A	29.7 ( ± 9.7)	N/A	N/A	N/A	N/A	31.1 (N/A)	N/A	N/A	N/A	N/A	64 (N/A)	N/A	48.7 ( ± 15.9)	N/A
Recipient	44.7 ( ± 12.8)	43.1 ( ± 8.7)	45.3 ( ± 11.1)	11.7 ( ± 5.5)	N/A	N/A	N/A	N/A	16.6 (N/A)	N/A	N/A	N/A	N/A	62.5 (N/A)	N/A	49.2 ( ± 14.9)	<18
**Acute rejection**	*n = 7*	*n = 7*	*n = 14*	*n = 18*	*n = 76*	*n = 24*	*n = 122*	*n = 78*	*n = 17*	*n = 14*	*n = 14*	*n = 83*	*n = 6*	*n = 4*	*n = 35*	*n = 56*	*n = 7*
*Donor type*																	
LD	1 (24%)	N/A	N/A	N/A	N/A	16 (67%)	N/A	N/A	N/A	N/A	N/A	N/A	N/A	1 (25%)	N/A	8 (14.6%)	N/A
DD	6 (86%)	N/A	9 (64.2%)	N/A	39 (51.3%)	8 (33%)	N/A	N/A	N/A	N/A	N/A	N/A	N/A	3 (75%)	N/A	47 (85.4%)	N/A
*Gender: male/female*																	
Donor	N/A	N/A	N/A	57%/43%	43%/57%	71%/29%	N/A	N/A	57%/63%	N/A	N/A	N/A	N/A	50%/50%	N/A	43.4%/56.6%	N/A
Recipient	71%/29%	75%/25%	70%/30%	61%/39%	67%/33%	59%/41%	N/A	N/A	28.6%/71.4%	N/A	N/A	N/A	N/A	75%/25%	N/A	55.4%/44.6%	N/A
*Age (years)*																	
Donor	N/A	36.3 ( ± 8.3)	N/A	27.1 ( ± 13.6)	N/A	33 (N/A)	N/A	N/A	26.6 (N/A)	N/A	N/A	N/A	N/A	44.5 (N/A)	N/A	47.6 ( ± 14.4)	N/A
Recipient	35.2 ( ± 12.8)	43.9 ( ± 10.9)	43.3 ( ± 10.3)	11.9 ( ± 25.2)	N/A	11.3 (N/A)	N/A	N/A	12.5 (N/A)	N/A	N/A	N/A	N/A	55.2 (N/A)	N/A	47.6 ( ± 14.6)	<18
*Rejection type*																	
TCMR	5 (71%)	N/A	10 (71.4%)	16 (88.8%)	N/A	24 (100%)	35 (28.7%)	32 (41%)	13 (76.4%)	N/A	14 (100%)	N/A	N/A	4 (100%)	2 (5.7%)	0 (0%)	5 (71.4%)
ABMR	0 (0%)	N/A	0 (0%)	N/A	N/A	0 (0%)	65 (53.2%)	40 (51.3%)	0 (0%)	N/A	0 (0%)	N/A	N/A	0 (0%)	15 (40%)	56 (100%)	2 (28.5%)
Borderline	2 (29%)	N/A	4 (28.6%)	N/A	N/A	0 (0%)	0 (0%)	0 (0%)	4 (23.5%)	N/A	0 (0%)	N/A	N/A	0 (0%)	11 (31.4%)	0 (0%)	0 (0%)
Mixed	0 (0%)	N/A	0 (0%)	N/A	N/A	0 (0%)	22 (18%)	6 (7.7%)	0 (0%)	N/A	0 (0%)	N/A	N/A	0 (0%)	7 (20%)	0 (0%)	0 (0%)

Dataset individual description relative to published year, platform, rejection type, and respective sample number for each group.

LD, live donor; DD, deceased donor; TCMR, T cell-mediated rejection; ABMR, antibody-mediated rejection; N/A, Not answered.

**Figure 1:**
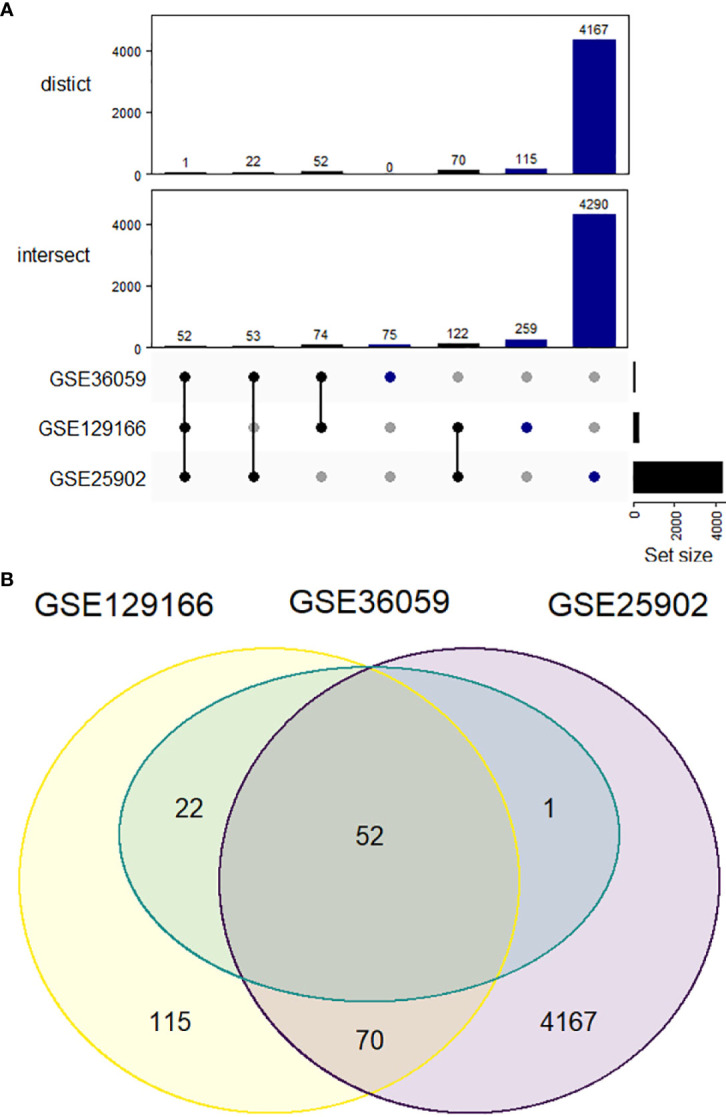
Upregulated differentially expressed genes comparing acute kidney rejection vs. stable graft. **(A)** Datasets represented by Venn diagram showing 52 overlapped genes between GSE25902, GSE36059, and GSE129166. **(B)** UpSetR representation of both intersect and distinct forms. The intersect method indicates whole gene similarity between compared studies, while distinct analysis plots the genes shared exclusively among each group of datasets.

In total, 52 genes were found commonly upregulated when comparing acute rejection to stable grafts (**Figure 1**). However, no overlapping could be defined for downregulated genes, which were found mostly differentially expressed in the GSE25902 dataset (6869 genes) but discreetly changed for GSE129166 (one gene, *RBP4*) and absent for GSE36059 (no genes).

### 3.2 Inflammatory and immune pathways are enriched upon graft rejection

**Figure 2:**
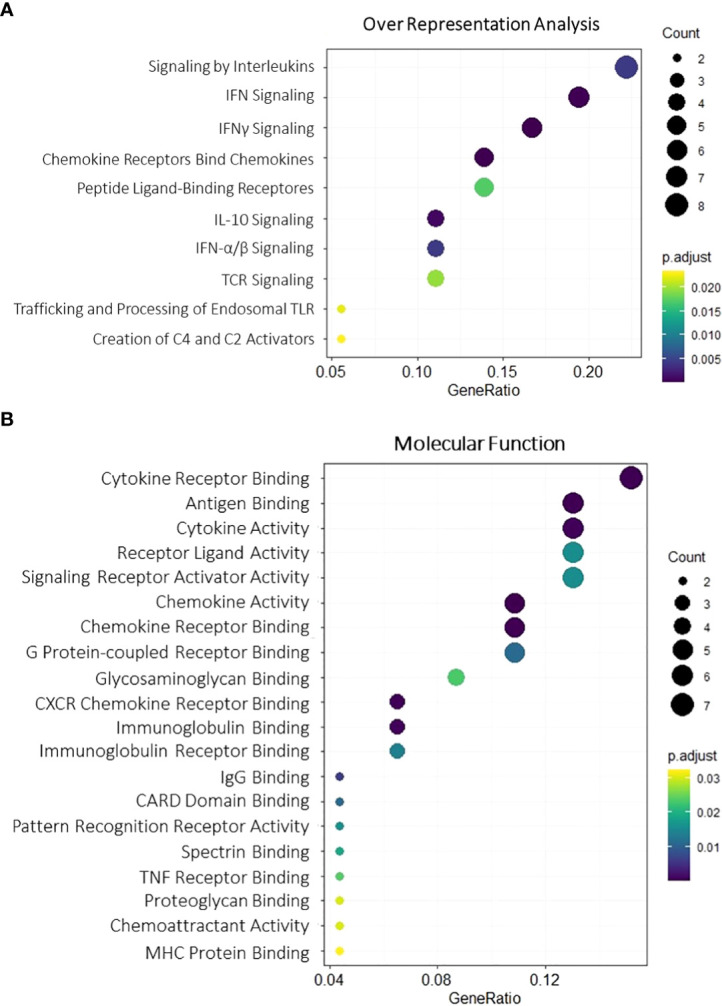
Gene Ontology enrichment for overlapping upregulated DEGs. Molecular function **(A)** and overall representation **(B)** for the 52 overlapping upregulated genes. Dot size is proportional to gene count, and color gradients follow adjusted p-value range, assuming higher significance for darker tones and lower significance for light ones. All terms were filtered for adjusted p-value < 0.05. DEGs, differentially expressed genes.

To better discern roles underlying the 52 upregulated genes, GO enrichment analysis was employed for Biological process, Molecular function, and Cellular components, revealing majorly immune-related terms (**Figure 2**) (**Supplementary Figure 2**) including interferon signaling (GO:0034341 GO:0034341), T-cell response (GO:0051251, GO:0042098), and CARD-binding domains (GO:0050700), processes that are in line with previous studies results showing both lymphocyte and interferon-gamma enhancement upon kidney rejection (38, 39). Potential upstream targets of the DEG list were concomitantly accessed by the X2K tool and indicated consistent Mitogen-activated protein kinases enrichment (*MAPK1*, *MAPK8*, *MAPK9*, *MAPK10*, and *MAPK14*) and transcription factors befitting inflammatory and lymphocyte pathways (*TBX21*, *SP100*, *IRF8*, and *BATF*) activation (**Supplementary Figure 3**), highlighting a possible role for these mechanisms in the context of acute rejection.

### 3.3 Inflammasome expression is enhanced upon rejection and correlates with lymphocyte populations

**Figure 3:**
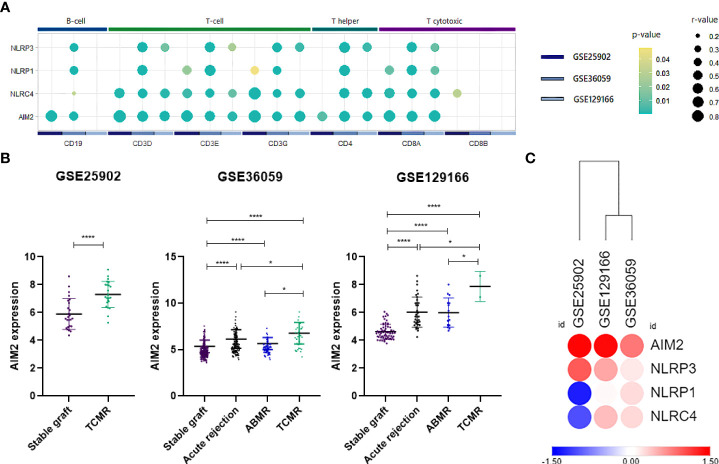
AIM2 and inflammasomes expression. **(A)** Log_2_ fold-change heatmap for inflammasomes comparing acute rejection samples to stable grafts. **(B)** Scatter plot of AIM2 distribution for three selected datasets (GSE25902, GSE36059, and GSE129166), considering overall acute rejection and its subdivisions TCMR and ABMR. **(C)** Summarized dot plot representing inflammasome correlations between lymphocyte surface markers. Size is designed proportionally to r-value. Yellow color is attributed to less significant correlations, and green tones are attributed to more significant ones. Correlations that did not reach a p-value <0.05 were omitted from the scheme. TCMR, T cell-mediated rejection; ABMR, antibody-mediated rejection.

From the previous analysis, we identified CARD-binding domains as enriched terms. CARD activation is traditionally reported on direct or indirect caspase-1 recruitment by inflammasomes (40). In this sense, we first evaluated whether the classically studied inflammasomes NLRP1, NLRP3, NLRC4, and AIM2 could participate in acute rejection. Accordingly, our representative heatmap of the GSE25902, GSE36059, and GSE129166 datasets showed reproducibility and increased expression, especially of *AIM2* and *NLRP3* (**Figure 3A**). Also, scatter plot visualization discerns that this enhancement was significant for *AIM2* in TCMR (MD = 1.401, 1.426, and 3.248, p < 0.0001) and ABMR (MD = 1.113 and 1.848, p < 0.0001) when compared to stable grafts, with TCMR reaching statistically higher levels among rejection groups (**Figure 3B**).

Since the products of inflammasome activation, such as cytokines and antigens, have been shown to provide signals for T-cell activation, serving as potential interfaces between innate and adaptive immunity (41, 42), we therefore performed correlations between inflammasome genes and surface markers of T cells and B cells. Our analysis outlined a positive association mainly between *NLRC4* and *AIM2* for *CD3*, *CD4*, and *CD8A*. Additionally, *NLRP1* and *NLRP3*, albeit attained fewer and weaker correlations, were still significant for major T-cell molecules (**Figure 3C**) (**Supplementary Figure 4**). Given that *AIM2* presented the most significant correlations with T-cell populations, we then hypothesized that AIM2 inflammasome could play a significant role in the regulation of T lymphocytes during allograft rejection.

### 3.4 AIM2 is associated with allograft rejection and oxidative phosphorylation gene sets

To better understand the role of AIM2 in acute rejection and cellular dysfunction, we performed a GSEA (43) using the three initial datasets to compare the *AIM2* phenotype with *Hallmarks* gene sets. The analysis indicated allograft rejection, interferon, and inflammatory response as the main processes positively regulated for the *AIM2* phenotype, while oxidative phosphorylation (OXPHOS) and fatty acid metabolism were most negatively regulated (**Supplementary Table 1**). When comparing acute rejection to stable graft phenotype, similar results were reproduced, particularly concerning positive processes and the OXPHOS enrichment in non-rejected kidneys (**Supplementary Table 2**).

**Figure 4:**
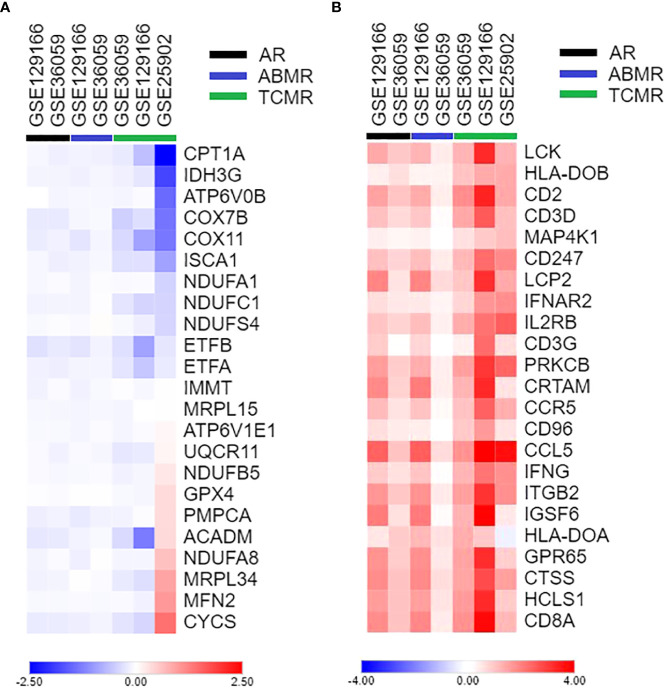
Heatmap of differentially expressed genes between acute rejection and stable graft groups. **(A)** Top 10 genes of allograft rejection GSEA gene set and inflammasomes. **(B)** Top 10 genes of oxidative phosphorylation GSEA gene set and inflammasomes. GSEA, Gene Set Enrichment Analysis.

**Figure 5:**
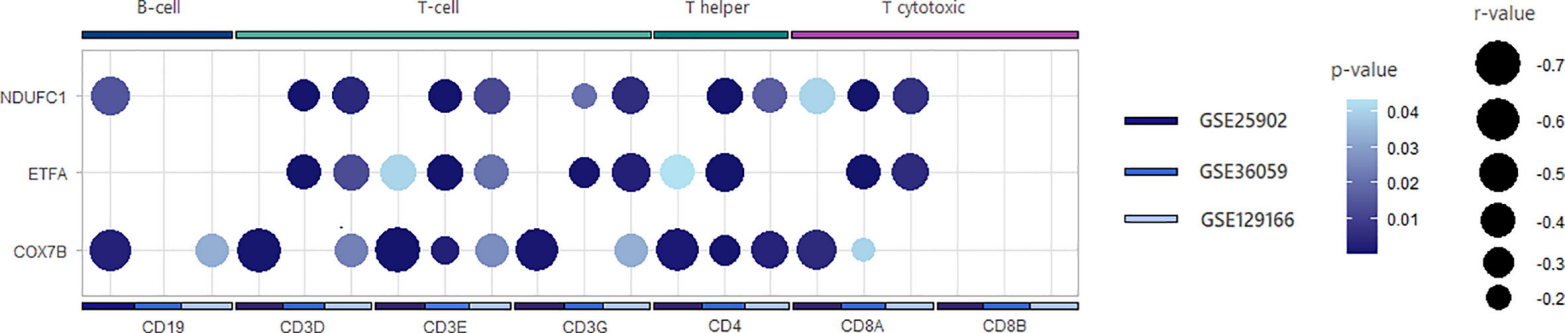
Correlation plot between oxidative phosphorylation genes and lymphocyte receptors genes. Summarized plot representing correlations’ magnitude and significance. Dot size is inversely proportional to r coefficient. Dark blue color indicates more significant correlations, while light tones indicate less significant p-values. Correlations that did not reach p-value <0.05 were suppressed from the plot.

Considering that allograft rejection and OXPHOS were the main regulated pathways for both the *AIM2* phenotype and acute rejection group and that the current literature supports intracellular metabolism as a potential driver of different leukocytes responses (23), we aimed to access how the central genes of these processes were expressed according to rejection type and lymphocyte infiltration. For that, the top 10 most enriched genes linked to allograft rejection or OXPHOS were selected for a log_2_FC heatmap (**Figure 4**) (**Supplementary Figure 5**), which demonstrated a reproductive different expression, mainly on TCMR subgroups. When analyzing associations to lymphocyte markers, the OXPHOS genes *NDUFC1*, *ETFA*, and *COX7B* were the most negatively correlated to CD3 subunits, *CD8A* and *CD4* (**Figure 5**) (**Supplementary Figure 6**), suggesting that a decrease in OXPHOS could either arose from the metabolic shift in T-cell populations or be influenced by these cells’ infiltration.

### 3.5 AIM2 is accurate in discerning acute rejection grafts and implies lower graft survival

**Figure 6:**
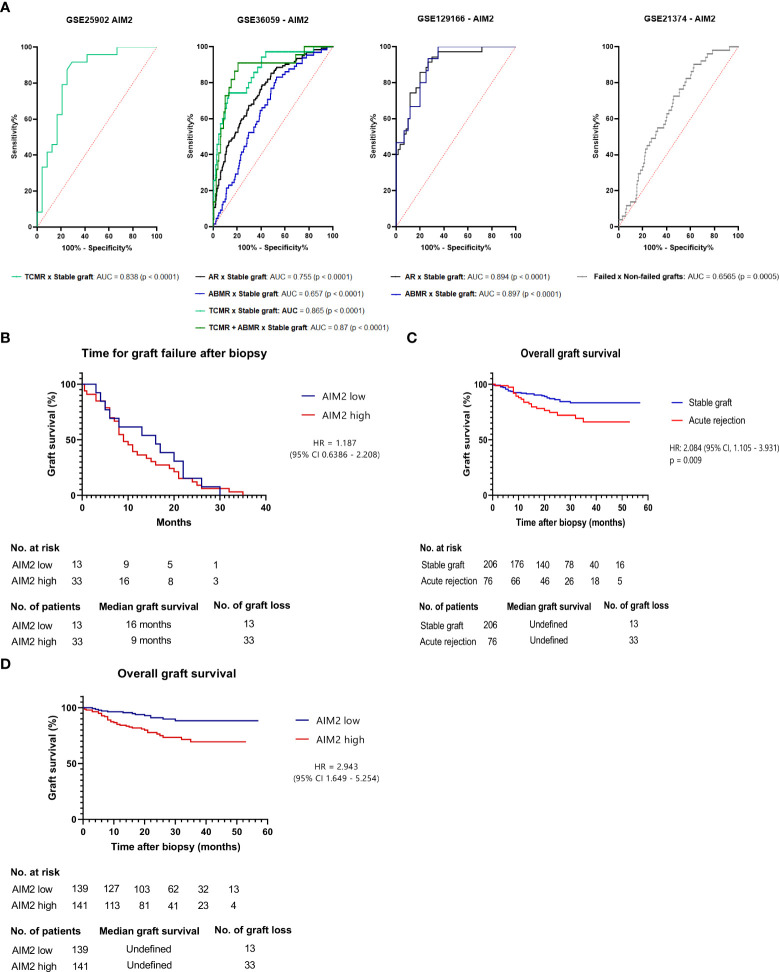
Clinical outcomes linked to AIM2 **(A)** Receiver Operating Characteristic Curve (ROC) depicting AIM2 accuracy for GSE25902, GSE36059, GSE129166, GSE21374 and its respective TCMR or ABMR samples **(B)** Graft survival for GSE21374 stable graft and acute rejection samples predicted by Kaplan-Meier curve on log-rank (Mantel-Cox) test. Groups were separated in low or high AIM2 levels according to a 5.738 cut-off that showed 78.95% sensitivity and 71.84% specificity for acute rejection prediction on ROC curve **(C)** Graft survival for acute rejected samples of GSE21374, applying same 5.738 threshold to determine AIM2 high or low groups. Statistics were determined by the log-rank test **(D)** Time for graft failure after biopsy for GSE21374 dataset.

After discerning potential molecular processes aligned to *AIM2*, we enquired whether this gene could reach clinical relevance in identifying acute rejection grafts. In this sense, we employed ROC curves for GSE36059, GSE129166, and GSE25902 (**Figure 6A**), indicating global accuracy to discern overall rejections (area under the curve (AUC) = 0.755 and 0.894, p < 0.0001) possibly in a more pertinent fashion for TCMR (AUC = 0.865, p < 0.0001) than for ABMR (AUC = 0.657, p < 0.0001), even though just a single dataset enabled their simultaneously comparison.

To investigate if patients’ outcomes could also be influenced by the gene, an additional dataset (GSE21374) was used to assess graft survival rate based on pre-failure *AIM2* expression—this dataset was not added in the previous genome analysis due to the fact that it investigated gene expression prior to transplant; hence, it could not be included in the analysis of “acute rejection versus no-rejection”. Interestingly, we found that *AIM2* is indeed useful to determine failure susceptibility even in biopsies taken before graft dysfunction (AUC = 0.6565, p = 0.0005) (**Figure 6A**). In this line, a value comprising 72.55% sensitivity and 54.11% specificity for failure risk was defined to classify grafts in *AIM2-*high or *AIM2-*low expressions, in order to evaluate long-term graft survival. Accordingly, the Kaplan–Meier curves for both groups demonstrated that higher AIM2 grafts achieved shorter survival periods than the lower-expression group (hazard ratio (HR) = 2.943, 95% CI = 1.649–5.254, p < 0.0005) (**Figure 6B**). Interestingly, this survival difference was more pronounced and statistically significant than the one measured between acute rejection and stable grafts (HR = 2.084, 95% CI = 1.105–3.931 p < 0.009) (**Figure 6C**), suggesting that *AIM2*-related failure could potentially act regardless of rejection development. However, when a similar curve is performed including only thefailed grafts, no difference could be observed in survival ratesaccording to *AIM2* expression (HR = 1.187, 95% CI = 0.6386–2.208, p = 0.5813) (**Figure 6D**), indicating that *AIM2* is possibly involved in triggering graft loss, but not on its failure time course.

### 3.6 Meta-analysis for AIM2 expression supports its major enhancement on T cell-mediated rejection grafts

**Figure 7:**
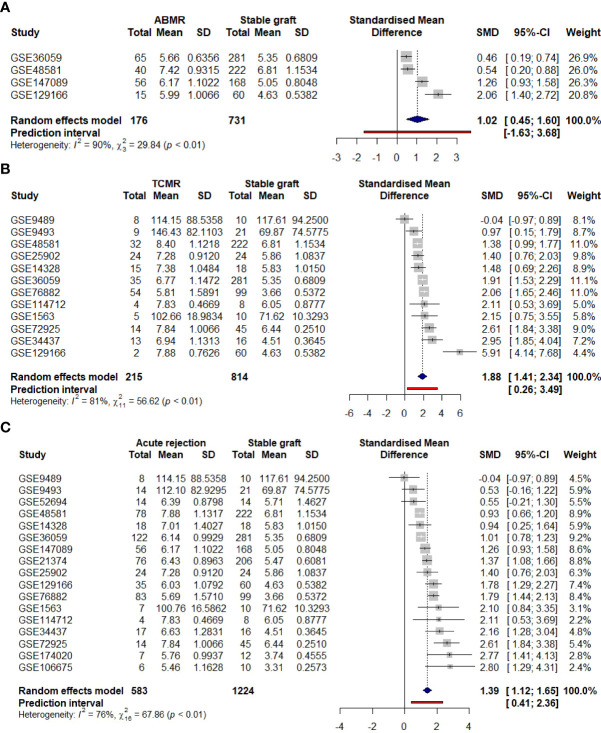
Clinical meta-analysis for *AIM2* employing a random-effects model. Lines correspond to 95% confidence intervals. Square size is proportional to study weight on overall effect. Right axis indicates AIM2 enhancement on acute rejection, while left axis indicates an elevation of stable graft. **(A)** General comparison considering all acute rejections and stable graft samples. **(B)** Subgroup analysis of the 11 TCMR-only grafts. **(C)** Subgroup analysis of the four ABMR-only grafts. TCMR, T cell-mediated rejection; ABMR, antibody-mediated rejection.

To support data confidence, a meta-analysis was conducted for 15 previously selected studies of either TCMR, ABMR, or borderline patients, totalizing 575 acute rejection samples and 1,214 stable grafts. The result was calculated for the SMD variable and selecting the random-effects model, due to expected differences in rejection types and demographics, features that were not standardized in the dataset description. Indeed, primary analysis showed considerable *AIM2* enhancement upon rejection (SMD = 1.45, [CI 95%, 1.18 to 1.71]) followed by significant high heterogeneity (*I* ^2^ = 75%, p < 0.01) (**Figure 7A**). Subgroup analysis indicated a higher to *AIM2* upregulation on TCMR (SMD = 2.01, [CI 95%, 1.58 to 2.45]) when compared to ABMR (SMD = 1.02, [CI 95%, 0.45 to 1.60]), although maintaining an elevated heterogeneity (TCMR *I* ^2^ = 76%, p < 0.01; ABMR *I* ^2^ = 90%, p < 0.01) (**Figures 7B, C**).

To access each study collaboration on the computed heterogeneity, an additional test was performed on the 15 datasets with *Influence Analysis*, which re-calculates *I* ^2^ values excluding one of the initial datasets. For the overall meta-analysis, GSE72925 and GSE76882 showed major contributions to heterogeneity, while for either TCMR or ABMR subgroups, GSE129166 was the main outline. Nevertheless, even with the respective studies’ omission, heterogeneity could just slightly improve, maintaining a high *I* ^2^ value of 71%, 58%, and 87% (**Supplementary Figures 7A-C**).

## 4 Discussion

Acute rejection is a life-threatening condition affecting nearly 10% of kidney transplants of which 25% do not recover previous graft function (44, 45). Despite the improvement of immunosuppression, the currently available drugs show limitations in reducing these statistics, possibly due to targeting canonical pathways of lymphocyte activation, such as the mammalian target of rapamycin (mTOR), antigen presentation, and IL-2 transcription (46, 47). In this sense, the investigation of new processes involved in pathogenesis could act as adjuvant therapies, for either acute rejection prevention or treatment.

Here, using a transcriptomics-based approach, we innovatively suggest that inflammasomes could act as a complementary branch, reporting for the first time their enhancement and reproducibility on acute rejection, mainly for TCMR. Indeed, inflammasomes are known to be involved in renal homeostasis and inflammatory response (48, 49). Curiously, while NLRP3 has been the one most described in kidney acute and chronic injuries (48), we showed that its upregulation is less remarkable in acute rejection when compared to NLRC4 and AIM2. We hypothesize that this may reflect either the relevance of particular signals, for example, an NLRC4 activation by interferon-regulated genes (50), since interferon processes were enriched in our analyzed datasets, or an AIM2 stimuli secondary to dsDNA release, possibly donor-free DNA molecules (51–54) since apoptotic and necrotic cells are proven to be present in these grafts (55, 56).

Few studies have explored the association between inflammasome activation and the regulation of adaptive immunity. In this sense, it has been demonstrated that activated T cells display a complement C5 protein-dependent NLRP3 inflammasome assembly, which induces T helper 1 (Th1) responses (57), which are typically associated with autoimmune and infectious diseases. However, our group demonstrated that a gain of function in NLRP3 specifically in CD4+ T cells improved the clinical course of disease in a model of experimental autoimmune encephalomyelitis (EAE), which was associated with a reduction of Th1 and Th17 inflammatory responses and a shift toward a Th2 pattern (58). It is important to point out that inflammasome receptors can also modulate adaptive immune responses regardless of their canonical roles in inducing the assembly of the complexes. Accordingly, NLRP3 has been shown to translocate to the nucleus of Tregs and negatively regulate this population’s master transcriptional regulator *FOXP3*, impairing their differentiation efficiency (59). AIM2, in turn, has been shown to enhance the stability of Tregs through metabolic reprogramming toward beta-oxidation in the mitochondria, restraining autoimmune responses in an inflammasome-independent fashion (60). Altogether, these data indicate that inflammasome receptors are important players in the regulation of T-cell responses and suggest that these processes are context-dependent. Our results here provide evidence that AIM2 expression correlates positively with T cells and is linked to allograft rejection, highlighting a detrimental role for this molecule in the context of kidney transplantation, which could be better explored and targeted in further studies.

**Figure 8:**
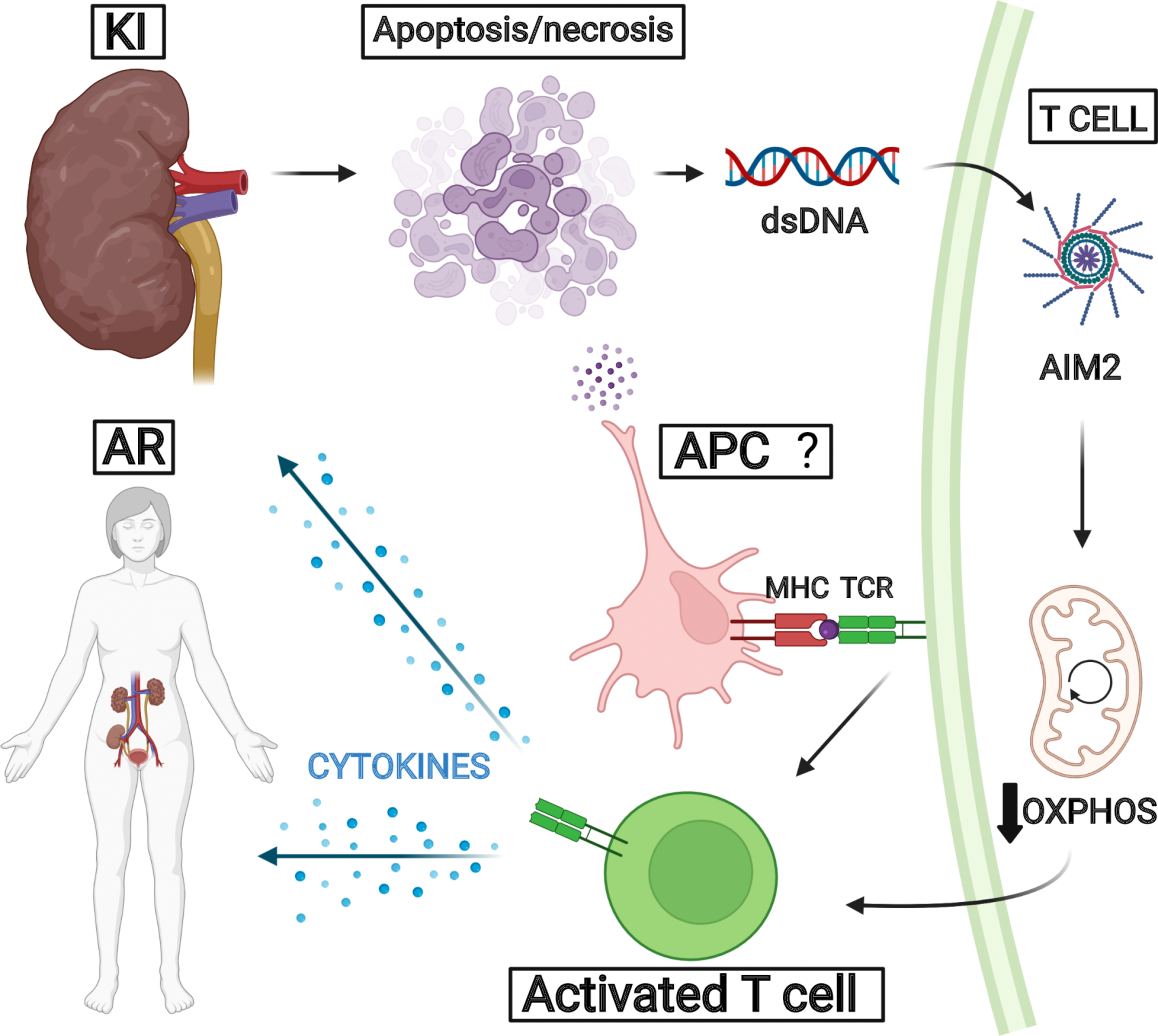
Proposed pathway for *AIM2* in acute rejection. We hypothesize that kidney injury secondary to transplantation could trigger tissue apoptosis and necrosis, leading to donor dsDNA release. This molecule could be recognized by AIM2 collaborating for T-cell activation related to OXPHOS decrease. The inflammatory processes reinforce kidney injuries and favor acute rejection development.

Additionally, T cells typically undergo metabolic rewiring after stimulation. The interplay between cell metabolism and effector immune function sets an emerging field termed “immunometabolism” (23), which is unraveling mechanisms that may propose promising therapeutic targets to dampen allograft rejection. Th1 and Th17 cells mostly rely on glycolytic activity in order to shape an effector response, and the MTORC1 and HIF-1α transcription factor are upregulated in both of these cells. However, Tregs usually activate the AMPK pathway, leading to lipid oxidation, OXPHOS, and suppression of immune responses (21, 22). In our results, the enrichment analysis showed that OXPHOS was negatively correlated to *AIM2* expression and rejected grafts. We also observed that OXPHOS-related genes were negatively correlated to lymphocyte markers such as CD3 and CD4. Studies show that pharmacological glycolysis ablation can enhance levels of CD4+Foxp3+ cells and reduce CD8+IFN-γ+ cells in transplanted mice (25). Also, in graft-*versus*-host disease (GvDH), when T cells encounter alloantigens, they preferentially upregulate glycolytic metabolism to produce pro-inflammatory responses. Ablation of glycolysis activator MTORC1 alleviated GvDH progression in mouse models (61). Although our results do not directly provide evidence for different T lymphocyte subset metabolic profiles, they suggest a correlation with these studies in a way that T cells present in rejected kidney allografts may have reduced levels of OXPHOS and lipid oxidation, which may enhance their inflammatory phenotype and, therefore, increase transplant rejection. A proposed integrative mechanism for AIM2 based on our findings is demonstrated in **Figure 8**.

Finally, meta-analysis is a strategy to quantify a combined effect size of different studies (62), in the case of bioinformatics, usually employing the standardized mean difference (SMD) variable, which was also applied to remove batch effects, in order to reduce heterogeneity (63–65). In our data, AIM2 attained a significant SMD in the TCMR subgroup, in accordance with our individual-dataset heatmap, suggesting a probable confident non-random finding. Since demographic information is usually incompletely available either in the GEO platform or in published transcriptome papers, our analyzed microarrays could have embraced grafts from different donor types, distinct ischemia periods, and immunosuppressive schemes, which are conditions that affect acute rejection development, potentially modifying our measured effect (66–68). In spite of diagnostic heterogeneity, expression profiling by array has intrinsic variations that may also contribute to this observation. It has been noted that microarrays carried out at different time points but performed in the same laboratory still show discrepancies among them (69). Since the datasets analyzed in our work were retrieved from different centers, we standardized the type of annotation that the microarray was provided, which aids in reducing batch effects (69, 70). In this line, further clinical studies are desired to overcome the intrinsic heterogeneity of *in silico* studies (65), supporting and refining information concerning which grafts are more susceptible to AIM2 signaling and, thus, identifying the patients who could benefit from drug trials targeting the inflammasomes as adjuvant therapies (71–73).

Currently, acute rejection is suspected with parameters for acute kidney injury—rise in 48 h of serum creatinine >0.3 mg/dl or >50% or oliguria < 0.5 ml/kg/h for more than 6 h, with the diagnosis being made by the realization of biopsy to directly identify allograft rejection (74, 75). These criteria, however, present some clinical challenges: serum creatinine is not specific for allograft rejection and can be elevated in both acute rejection and progression of the disease or new pathologic processes in the kidney (76). Furthermore, the confirmation *via* kidney biopsy brings financial limitations—since it is necessary for the patient to be hospitalized—as well as the bias of sampling error or variability between professionals (77). This context led to the search for non-invasive biomarkers that could predict acute rejection, specifically in blood and urine samples that were linked to TCMR, like CXCL10, TNF-alpha, and miR-155-5p (74, 75). Our findings for AIM2 expression in the tissue and accuracy for graft failure could prompt new analysis for its expression in patient biosamples.

Even though AIM2 has been in recent discussions as a potential target to understand and regulate the progression of inflammatory conditions (78), we were unable to find references that directly linked the molecule to acute rejection both inside and outside the context of a kidney transplant. Going further from the potential mechanism linked to AIM2 and acute rejection previously discussed, markers that permit identifying and predicting acute rejection in kidney transplants are still being researched. In summary, potential non-invasive markers bring a new perspective to the diagnosis of acute rejection as well as a potential way of introducing different interventions according to the findings. In this sense, AIM2 could be a potential new biomarker, especially for TCMR. Even though the previously cited markers showed a good overall diagnostic accuracy with a more practical clinical approach since they are collected from the blood and urine; for acute rejection, here we also include the potential use of AIM2 as a predictor for overall graft survival and not only acute rejection. This could prompt the evaluation of AIM2 as a marker in blood and urine as well.

## Data availability statement

The datasets presented in this study can be found in online repositories. The names of the repository/repositories and accession number(s) can be found in the article/**Supplementary Material**.

## Author contributions

NF and JV selected datasets and wrote the first draft of the manuscript. LE and BG contributed writing sections of the manuscript. NF and LE performed computational analysis. NF, JV and BG designed correlations. NF, JV, LE, IZ, GR and BG collaborate in data interpretation and visualization. NO conceived and supervised the study. All authors contributed to edition, critical revision and approved the submitted version.

## Funding

The research was supported financially by São Paulo Research Foundation (FAPESP) (18/21844-6, 2019/22409-4, 17/05264-7) and National Council for Scientific and Technological Development (CNPq) and CAPES (financial code 001) grants.

## Conflict of interest

The authors declare that the research was conducted in the absence of any commercial or financial relationships that could be construed as a potential conflict of interest.

## Publisher’s note

All claims expressed in this article are solely those of the authors and do not necessarily represent those of their affiliated organizations, or those of the publisher, the editors and the reviewers. Any product that may be evaluated in this article, or claim that may be made by its manufacturer, is not guaranteed or endorsed by the publisher.

## Supplementary material

Supplementary Figure 1 Query strategy. Flowchart for systematic studies selection, adapted from Reporting Items for Systematic Reviews and Meta-Analyses (PRISMA) 2020 guidelines.

Supplementary Figure 2 Transcription factor and kinase enrichment analysis. **(A).** The bar chart is representative of the mean rank of the highest ranked transcription factors from ChEA3. The y-axis displays the different transcription factors, and the x-axis displays the average mean rank of a transcription factor across all the available libraries. **(B).** The bar chart is a mean rank bar chart for the top ranked kinases from KEA3. The y-axis displays the different kinases, and the x-axis displays the mean rank of a specific kinase across all the available libraries.

Supplementary Figure 3 Gene Ontology enrichment for overlapping up-regulated DEGs. Biological processes and cellular components representation for the 52 overlapping up-regulated genes. Dot size is proportional to gene count, and color gradients follow adjusted p-value range, assuming higher significance for darker tones, and lower significance for light ones. All terms were filtered for adjusted p-value < 0.05.

Supplementary Figure 4 Correlation plot between lymphocyte receptors genes and AIM2 expression **(A)** CD3 subunits used as general T-cell markers **(B)** CD4 expression used as T-helper signature **(C)** CD8 subunits used to represent T-cytotoxic cells **(D)** CD19 expression used for B-cell assessment.

Supplementary Figure 5 Log-2-fold-change heatmap of differentially expressed genes between acute rejection and stable graft groups for all datasets (A) Top 10 genes of allograft rejection GSEA geneset and inflammasomes (B) Top 10 genes of oxidative phosphorylation GSEA geneset and inflammasomes.

Supplementary Figure 6 Correlation plot between lymphocyte receptors genes and oxidative phosphorylation related genes **(A)** CD3 subunits used as general T-cell markers **(B)** CD4 expression used as T-helper signature **(C)** CD8 subunits used to represent T-cytotoxic cells **(D)** CD19 expression used for B-cell assessment.

Supplementary Figure 7 Studies heterogeneity contribution accessed by *InfluenceAnalysis* **(A)** General comparison considering all acute rejections and stable graft samples **(B)** Subgroup analysis of the 11 TCMR-only grafts **(C)** Subgroup analysis of the 4 ABMR-only graft.

Supplementary Table 1 Genesets related to AIM2. Top 5 genesets of GSEA Hallmark enrichment for AIM2 phenotype, indicating processes more positively or negatively correlated to AIM2.

Supplementary Table 2 Genesets related to acute rejection. Top 5 genesets of GSEA Hallmark enrichment comparing acute rejection vs. stable graft phenotype.

Supplementary Table 3 Hallmarks positively and negatively enriched in acute rejection.
