# Supplementary figures and images for: AIM2 as a putative target in acute kidney graft rejection
Source: Front Immunol. 2022 Sep 30;13:839359. doi: 10.3389/fimmu.2022.839359 (PMC9561248; doi:10.3389/fimmu.2022.839359)

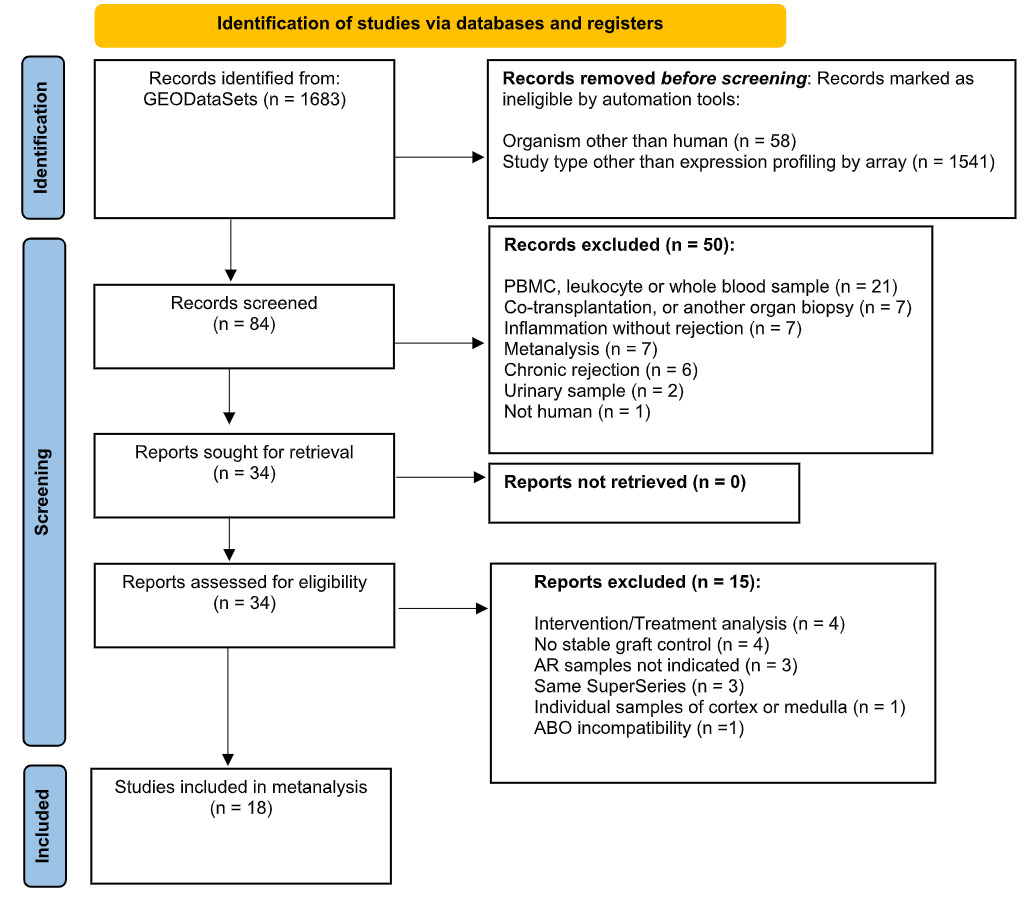

Supplement: Supplementary Figure 1 — Query strategy. Flowchart for systematic studies selection, adapted from Reporting Items for Systematic Reviews and Meta-Analyses (PRISMA) 2020 guidelines. [file Image_1.tiff]

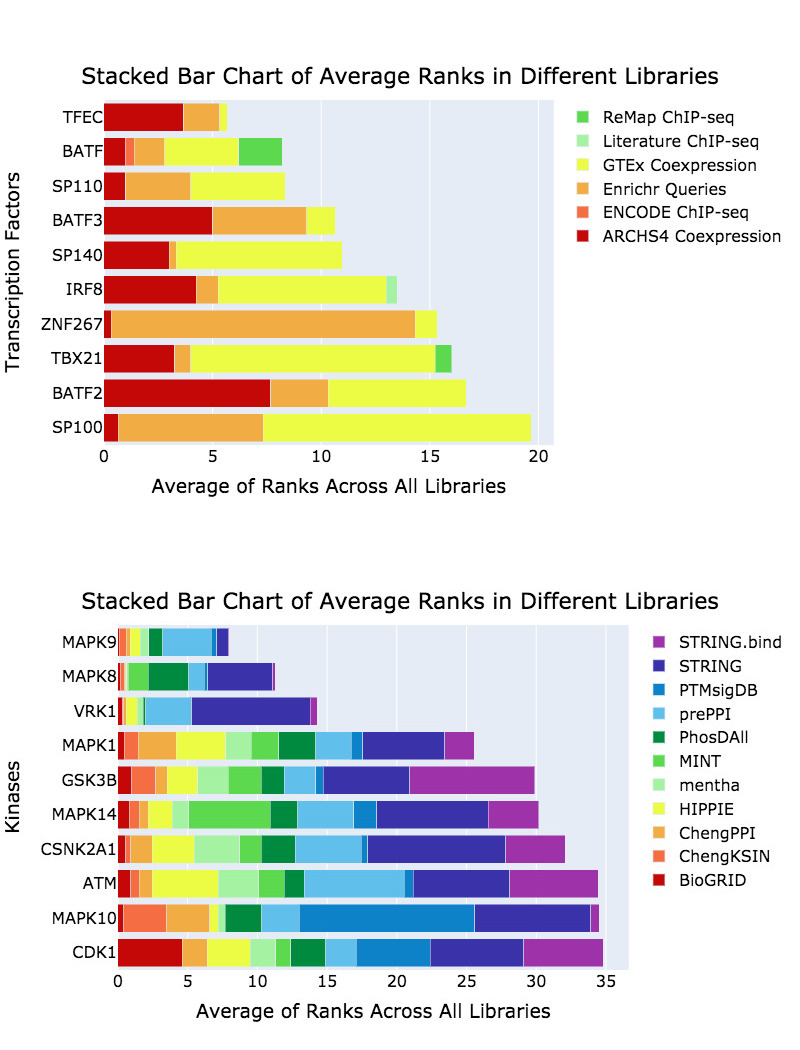

Supplement: Supplementary Figure 2 — Transcription factor and kinase enrichment analysis. (A). The bar chart is representative of the mean rank of the highest ranked transcription factors from ChEA3. The y-axis displays the different transcription factors, and the x-axis displays the average mean rank of a transcription factor across all the available libraries. (B). The bar chart is a mean rank bar chart for the top ranked kinases from KEA3. The y-axis displays the different kinases, and the x-axis displays the mean rank of a specific kinase across all the available libraries. [file Image_2.jpeg]

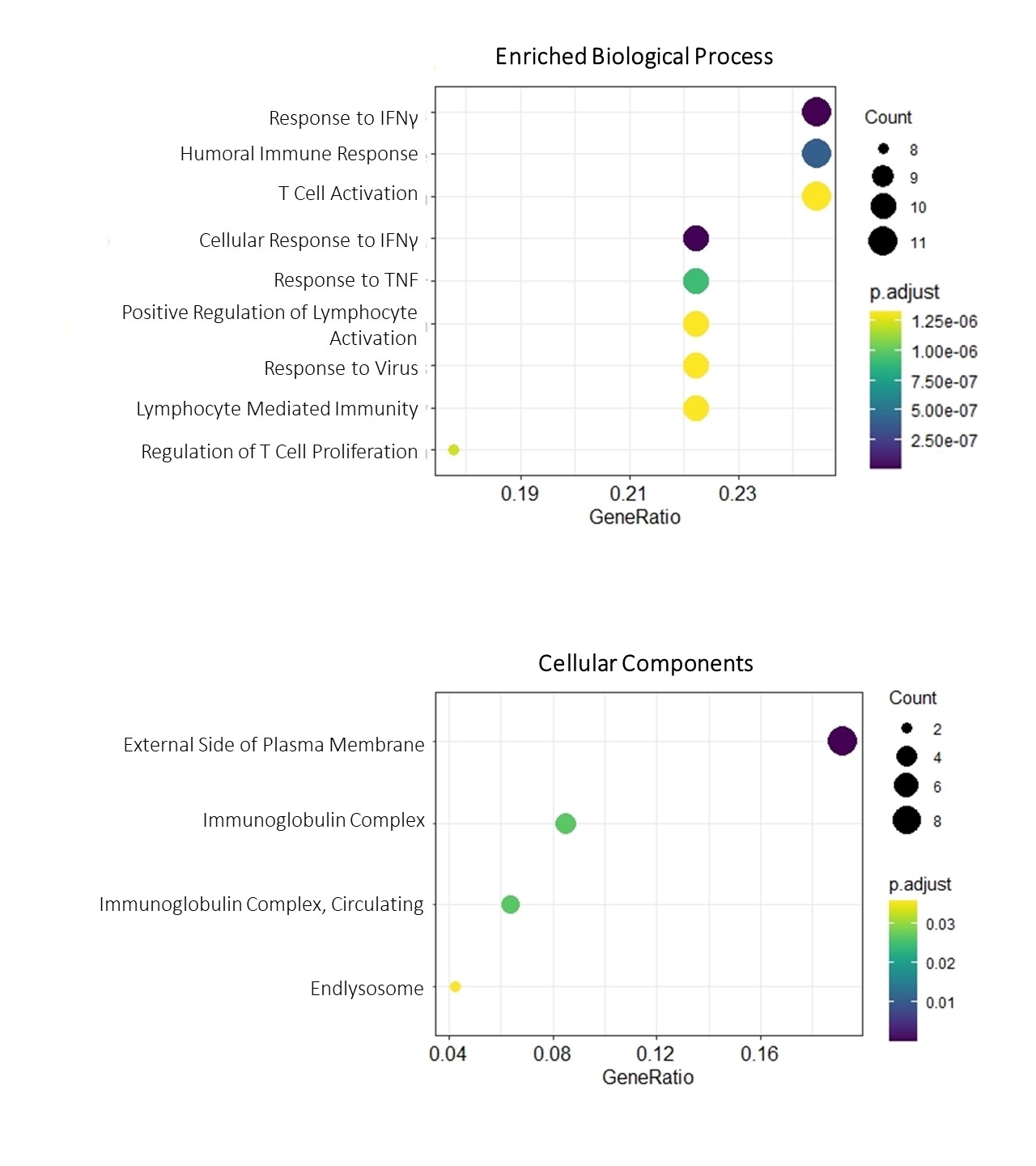

Supplement: Supplementary Figure 3 — Gene Ontology enrichment for overlapping up-regulated DEGs. Biological processes and cellular components representation for the 52 overlapping up-regulated genes. Dot size is proportional to gene count, and color gradients follow adjusted p-value range, assuming higher significance for darker tones, and lower significance for light ones. All terms were filtered for adjusted p-value < 0.05. [file Image_3.jpeg]

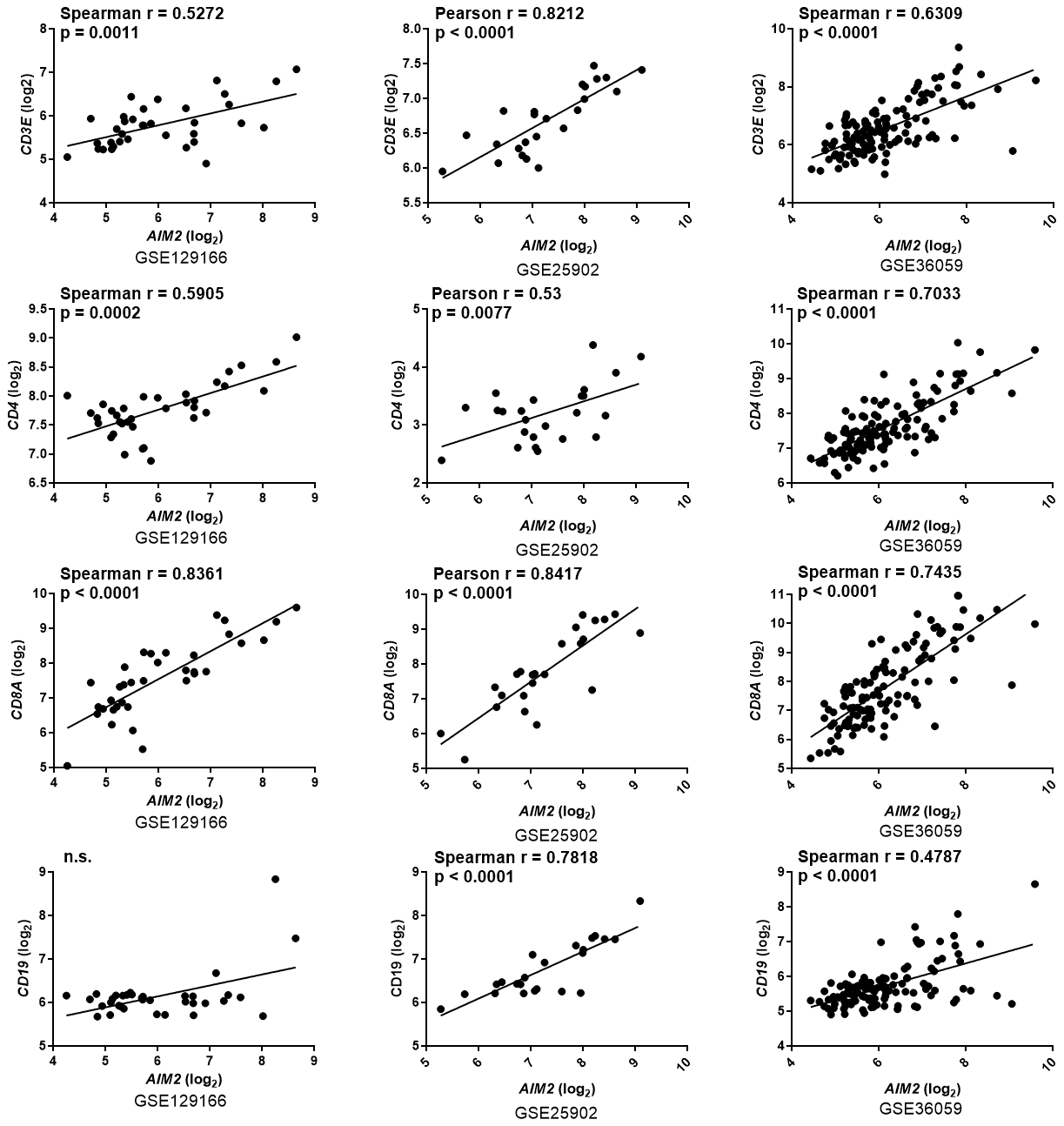

Supplement: Supplementary Figure 4 — Correlation plot between lymphocyte receptors genes and AIM2 expression (A) CD3 subunits used as general T-cell markers (B) CD4 expression used as T-helper signature (C) CD8 subunits used to represent T-cytotoxic cells (D) CD19 expression used for B-cell assessment. [file Image_4.jpeg]

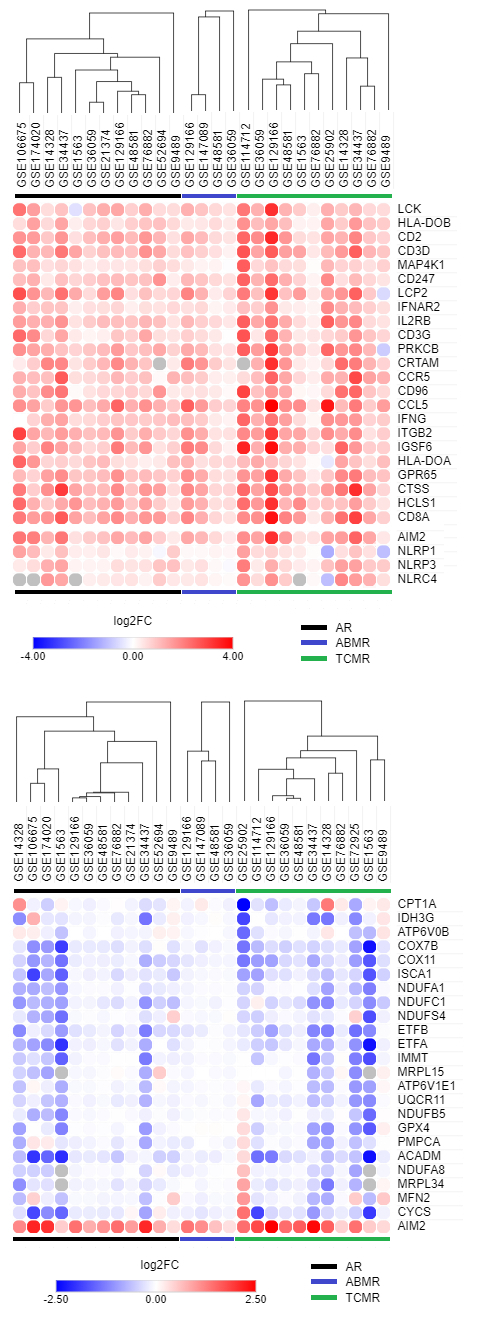

Supplement: Supplementary Figure 5 — Log-2-fold-change heatmap of differentially expressed genes between acute rejection and stable graft groups for all datasets (A) Top 10 genes of allograft rejection GSEA geneset and inflammasomes (B) Top 10 genes of oxidative phosphorylation GSEA geneset and inflammasomes. [file Image_5.jpeg]

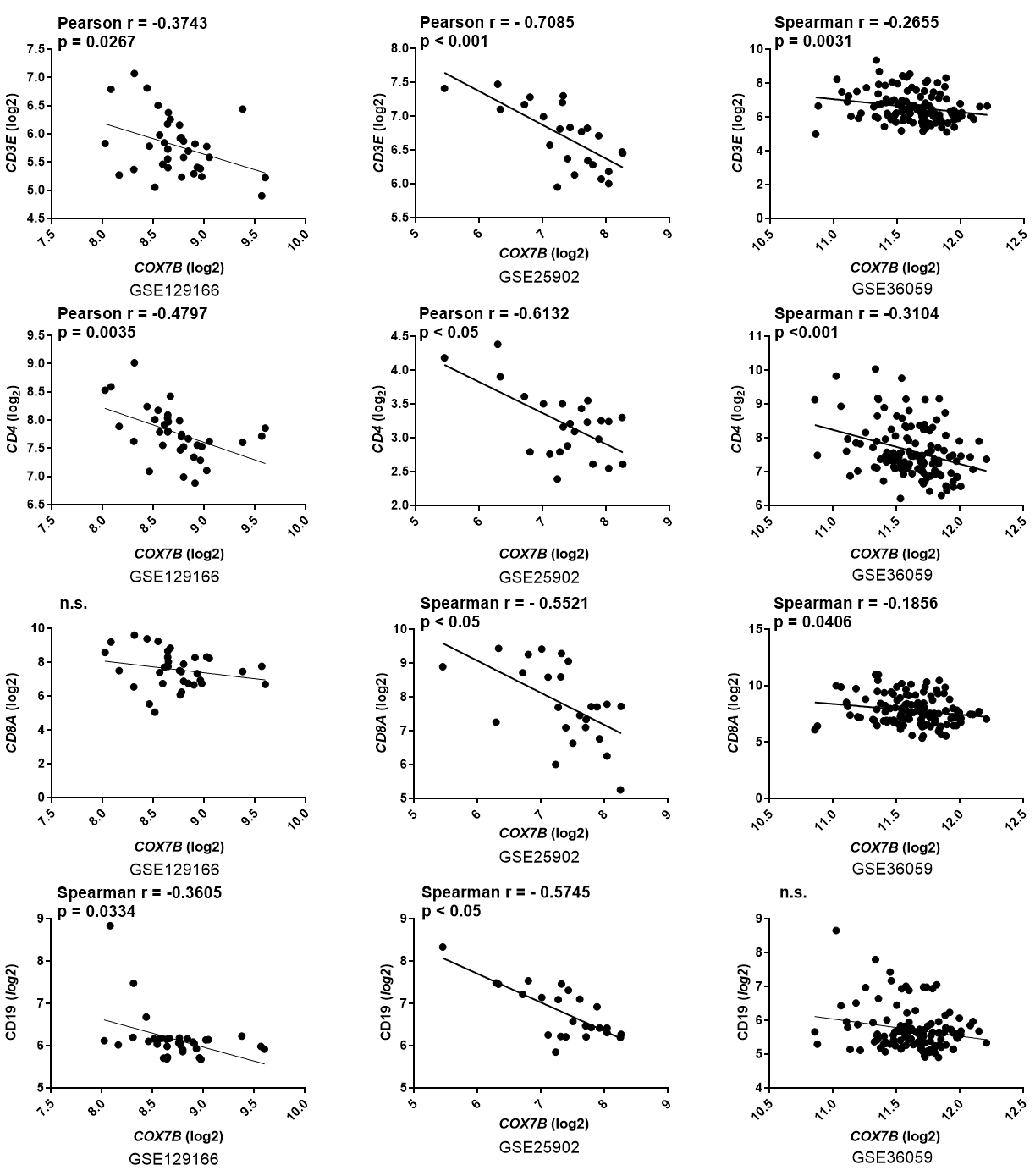

Supplement: Supplementary Figure 6 — Correlation plot between lymphocyte receptors genes and oxidative phosphorylation related genes (A) CD3 subunits used as general T-cell markers (B) CD4 expression used as T-helper signature (C) CD8 subunits used to represent T-cytotoxic cells (D) CD19 expression used for B-cell assessment. [file Image_6.jpeg]

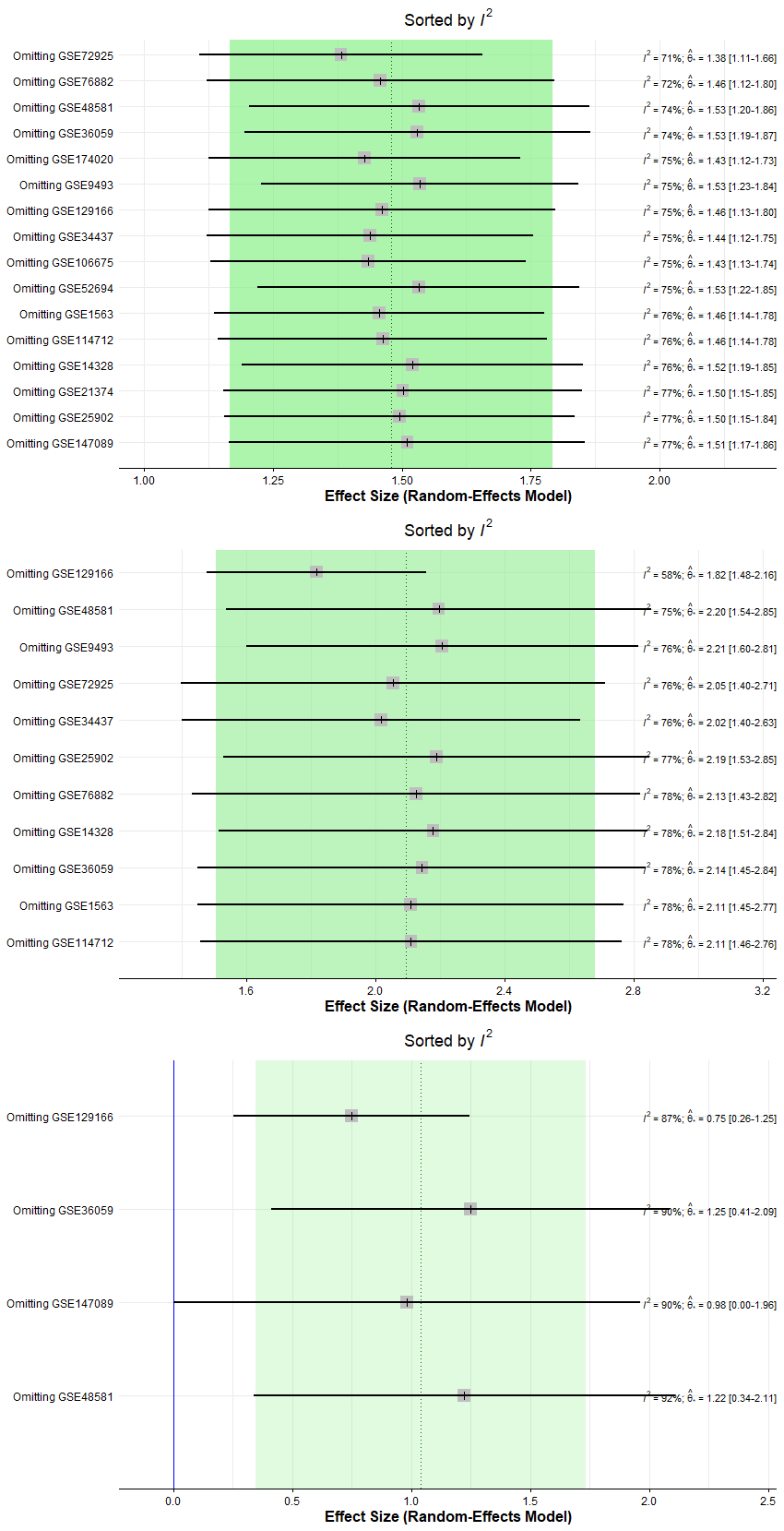

Supplement: Supplementary Figure 7 — Studies heterogeneity contribution accessed by InfluenceAnalysis (A) General comparison considering all acute rejections and stable graft samples (B) Subgroup analysis of the 11 TCMR-only grafts (C) Subgroup analysis of the 4 ABMR-only graft. [file Image_7.jpeg]
